# Letter to the editor: “Effects of exercise therapy on disability, mobility, and quality of life in the elderly with chronic low back pain—a systematic review and meta-analysis of randomized controlled trials”

**DOI:** 10.1186/s13018-024-04555-9

**Published:** 2024-01-20

**Authors:** Jianda Kong, Rao Fan, Lei Zhu

**Affiliations:** College of Sports Science, Qufu Normal University, Qufu, China

Dear Editor,

The purpose of this letter is to assist readers in gaining a more comprehensive understanding and clarification of some critical limitations present in the recently published paper in your esteemed journal, titled “Effects of exercise therapy on disability, mobility, and quality of life in the elderly with chronic low back pain: a systematic review and meta-analysis of randomized controlled trials” [1]. While this study offers valuable insights into the treatment of chronic low back pain in the elderly, there are significant limitations that may affect the generalizability and accuracy of its conclusions.

Firstly, there are some limitations in the database search extension of this study. Although it includes databases such as Web of Science, MEDLINE, Cochrane Library, CNKI, EMBASE, and PubMed, it overlooks other databases such as PsycINFO, Scopus, and CINAHL, as well as specialized databases in rehabilitation and sports medicine or clinical trial registries, such as ClinicalTrials.gov. This exclusion may lead to the omission of key studies, thereby affecting the comprehensiveness of the conclusions [2]. A study on the best combination of databases for literature retrieval in systematic reviews emphasized that certain databases are more effective in retrieving unique references than others, with databases such as EMBASE, MEDLINE, and Web of Science often retrieving the most unique references [2]. However, when the topic of review is directly related to its domain, specific subject databases are crucial [2].

Furthermore, this study has demonstrated limitations in its inclusion criteria. While it is based on the PICOS principle and explicitly defines the participants as older adults with CLBP, including randomized controlled trials, it may not have thoroughly described the specific type, frequency, and duration of interventions [3]. The treatment modalities of the comparison groups may not have been fully elucidated, and primary and secondary outcome measures may not have been comprehensively listed. These omissions may limit the transparency and applicability of the research findings. The study has acknowledged the importance of accuracy in search strategies, particularly for systematic reviews of observational data, which are often less precise and more difficult to narrow down the scope [3].

Lastly, this study has shown limitations in addressing heterogeneity and subgroup analysis. Although subgroup analysis was conducted to explore the impact of different types of exercises on outcome measurements, the study may not have adequately considered heterogeneity factors such as age, gender, and duration of disease, which could affect the accuracy and applicability of the conclusions. This is a crucial point as the effectiveness of intervention measures may differ significantly among different subgroups, and understanding these differences is essential for clinical practice [4].

We hope this letter will help readers to better understand the research background and limitations of the paper, thereby enabling a more comprehensive evaluation of its results and conclusions. This is crucial for the application of these findings in clinical practice. We once again express our sincere gratitude to the authors for their hard work and sincerely hope that this will be beneficial to the readers.

## Data Availability

Not applicable.
